# Astrocyte-derived VEGF increases cerebral microvascular permeability under high salt conditions

**DOI:** 10.18632/aging.103348

**Published:** 2020-06-22

**Authors:** Zhezhi Deng, Li Zhou, Yuge Wang, Siyuan Liao, Yinong Huang, Yilong Shan, Sha Tan, Qin Zeng, Lisheng Peng, Haiwei Huang, Zhengqi Lu

**Affiliations:** 1 Department of Neurology, The First Affiliated Hospital, Sun Yat-Sen University, Guangdong Provincial Key Laboratory of Diagnosis and Treatment of Major Neurological Diseases, National Key Clinical Department and Key Discipline of Neurology, Guangzhou 510080, China; 2 Department of Neurology, The Third Affiliated Hospital of Sun Yat-Sen University, Guangzhou 510080, China

**Keywords:** high salt, astrocyte, VEGF, cerebral microvascular permeability

## Abstract

Excess salt (NaCl) intake is closely related to a variety of central nervous system (CNS) diseases characterized by increased cerebral microvascular permeability. However, the link between a high salt diet (HSD) and the breakdown of tight junctions (TJs) remains unclear. In the present study, we found that high salt does not directly influence the barrier between endothelial cells, but it suppresses expression of TJ proteins when endothelial cells are co-cultured with astrocytes. This effect is independent of blood pressure, but depends on the astrocyte activation via the NFκB/MMP-9 signaling pathway, resulting in a marked increase in VEGF expression. VEGF, in turn, induces disruption of TJs by inducing phosphorylation and activation of ERK and eNOS. Correspondingly, the HSD-induced disruption of TJ proteins is attenuated by blocking VEGF using the specific monoclonal antibody Bevacizumab. These results reveal a new axis linking a HSD to increased cerebral microvascular permeability through a VEGF-initiated inflammatory response, which may be a potential target for preventing the deleterious effects of HSD on the CNS.

## INTRODUCTION

Salt (sodium chloride, NaCl) intakes far outweigh the recommended levels in modern diet [1, 2]. A high salt diet (HSD) has long been considered to cause multiple severe vascular complications by up-regulating blood pressure, especially in salt-sensitive patients [3, 4]. In addition, recent studies have suggested a link between HSD, and immune and inflammatory diseases [5–7]. The brain is the principle organ impaired by HSD, which has been associated with various cerebral vascular and immuno-inflammatory diseases, such as stroke and multiple sclerosis (MS) [8, 9]. In the central nervous system (CNS), the most important converging point of blood vessels and immunity is the blood-brain barrier (BBB), which acts as a restricted interface insulating the CNS parenchyma from the peripheral circulation [10]. Whether high salt conditions can disrupt the function of the barrier serves as a motivation for our studies.

Increased permeability of the BBB is an early and common manifestation of most CNS diseases. The disruption of the BBB integrity is one of the key events under inflammatory CNS conditions, including cerebral ischemic injury and CNS immune diseases [11, 12]. Astrocytes are widely involved in the establishment, maintenance, and repair of the BBB [13]. A positive link has been identified between reactive astrocytes and BBB breakdown, and the astrocyte-derived VEGF has been shown to drive the BBB disruption in CNS inflammatory diseases [14, 15]. Our previous study showed that astrocytes are activated by high salt conditions, and release various inflammatory cytokines including VEGF [16], which is not only the most potent pro-angiogenic factor, but also increases vascular permeability.

Due to the tight renal regulation of plasma electrolytes, long-term intake of large amounts of salt may not increase plasma sodium levels, but may accumulate in the human interstitium independently of the kidney. The sodium concentration in the human interstitium is approximately 40mM higher than in plasma [17]. Astrocytes are the most widely distributed mesenchymal cells in the brain; they have the ability to respond quickly to changes in the microenvironment [18, 19].

Here we report that long-term HSD enhances cerebral microvascular permeability as indicated by the down-regulation of tight junction (TJ)-associated proteins, without changing the blood pressure. Overexpression of astrocyte-derived VEGF disrupts the function of the tight junction. Conversely, antagonizing VEGF using anti-VEGF neutralizing antibody can alleviate this damage. A better understanding of the effects of salt in CNS disease may provide a new theoretical basis for limiting the salt intake.

## RESULTS

### High salt diet increases the cerebral microvascular permeability by down-regulating expression of tight junction (TJ) proteins

**Figure 1:**
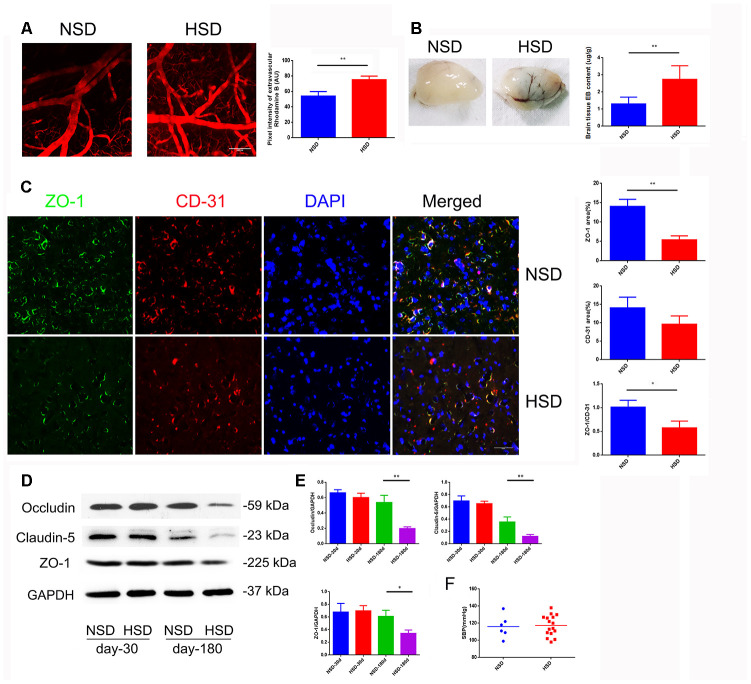
**HSD increases cerebral microvascular permeability.** (**A**) Representative images of the mice cerebral cortical microvessels and dye leakage at 10 min after injection of Rhodamine B isothiocyanate-dextran (n=3 per group). (**B**) Evans blue leakage analyzed in rats fed with selected diets for 180 days (n=3 per group). (**C**) ZO-1 (green) co-stained with CD31 (red), a microvascular endothelia marker, and DAPI (blue; n=6 per group) in the rats brain slices. (**D**, **E**) Expression of Occludin, Claudin-5 and ZO-1 in the brain tissues from rats analyzed by immunoblotting. (**F**) Systolic blood pressure (SBP) of rats fed with NSD or HSD for 180 days. *P<0.05, **P<0.01, compared with NSD; at least three separate experiments were conducted, means ± SD.

Increased cerebral microvascular permeability is the key to many peripheral inflammatory factors entering the CNS, leading to a variety of diseases. Inspired by previous epidemiological data, we studied the effect of HSD on the CNS. First, we analyzed the permeability of the mice cortical microvessels with a diameter of 20-40um *in vivo* by two-photon imaging. We found that intravascular dye leaked into extravascular space in the HSD group (Figure 1A). Next, we used the Evans blue dye (EBD) to assess the effect of HSD rats on the permeability of whole cerebral vessels. As shown in Figure 1B, the EBD leakage was significantly increased in the HSD group compared to the normal diet group (Day-180: *P*<0.05). Thus, we analyzed the expression of TJ-associated proteins in the brain tissues from rats fed HSD or normal salt diet (NSD), by using immunofluorescence staining and western blotting (Figure 1C, 1D). No significant changes were observed in the expression of ZO-1, occluding, and claudin-5 between the HSD and NSD groups, when high salt was fed for 30 days (Figure 1D, 1E). However, HSD dramatically decreased the expression of ZO-1, occludin, and claudin-5 after 180 days of high salt feeding (Figure 1D, 1E). Immunofluorescence double staining confirmed that the ZO-1 expression was down-regulated in CD31-positive microvascular in HSD rats fed for 180 days (Figure 1C).

Moreover, systolic blood pressure (SBP) was measured using a tail-cuff system in rats fed with normal or high-salt diet for 180 days. As shown in Figure 1F, there was no significant difference in the SBP between the normal and HSD groups.

### High salt activates astrocytes and increases expression of VEGF

**Figure 2:**
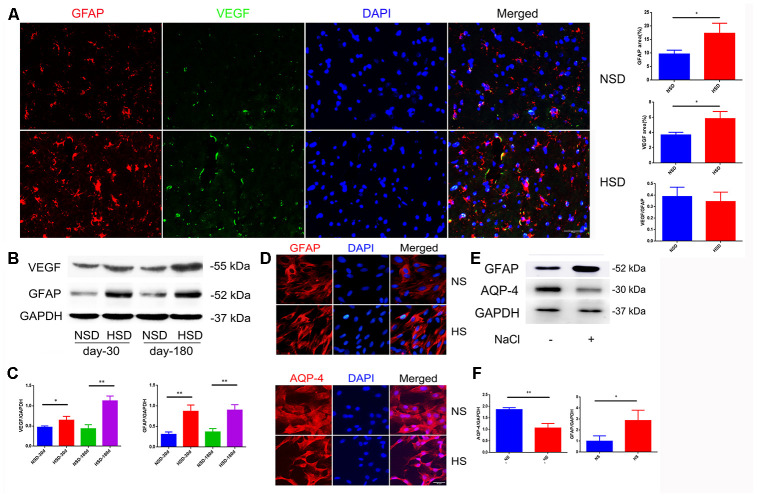
**High salt activates astrocytes and up-regulates VEGF.** (**A**) Representative images of double immunofluorescence staining for GFAP(red) and VEGF(green) in the brain specimens of rats; DAPI is stained in blue(n=5 per group). (**B**, **C**) Expression of GFAP and VEGF in the brain tissues from rats analyzed by western blotting and densitometry (n=5 per group). (**D**) Immunofluorescence staining of GFAP and AQP4 in red. (**E**, **F**) Expression of AQP4 and GFAP analyzed by western blotting in NaCl-treated astrocytes; *P<0.05, **P<0.01, compared with NS. At least three separate experiments were conducted; means ± SD.

Due to the strong effect on increased vascular permeability, VEGF is also called vascular permeability factor, which is mainly secreted by astrocytes [20]. Therefore, we examined the protein expression of VEGF and the astrocyte marker GFAP in the brain. Interestingly, immunofluorescence double staining showed that HSD up-regulated the expression of VEGF and GFAP in the rat brain (Figure 2A); this was also confirmed by western blotting (Figure 2B, 2C).

To investigate the effect of a high salt environment on astrocytes, primary astrocytes isolated from rat brain were treated with 40mM NaCl for 24h. Immunofluorescence staining and western blotting demonstrated an enhanced expression of GFAP (Figure 2D–2F). Aquaporin-4 (AQP-4) is a cell membrane protein that is highly expressed on the surface of astrocytes’ end-feet [21]. As shown in Figure 2D, the AQP4 expression on the end-feet changed from flat and uniform to slender and multipolar. Western blotting revealed that high salt treatment decreased the expression of AQP-4 (Figure 2E, 2F).

### High salt up-regulates expression of VEGF in astrocytes by activating NFκB/MMP-9 pathway

**Figure 3:**
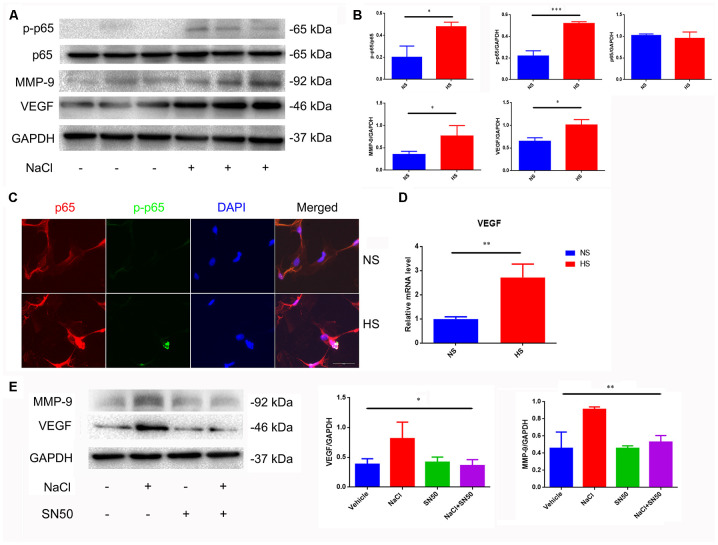
**Increased expression of VEGF is mediated by NFκB/MMP-9 pathway.** (**A**, **B**) HS increases protein levels of VEGF, MMP9 and p-p65. (**C**) Immunofluorescence staining of NaCl (40mM, 24 h)-induced p65 phosphorylation and nuclear translocation; (**D**) HS upregulates mRNA expression levels of VEGF. (**E**) SN50 antagonized NaCl-induced up-regulation of VEGF and MMP-9 protein expression. *P<0.05, **P<0.01, compared with NS, n=3, means ± SD.

Nuclear factor-κB (NF-κB) is a nuclear transcription factor that regulates the expression of pro-inflammatory genes [22]. The activation of NF-κB is considered to be part of a stress response as it is activated by a variety of stress stimuli. Phosphorylation of p65 NF-κB subunit and its nuclear translocation reflect the degree of NF-κB activation [22]. Our results show that the ratio of p-p65/p65 increases and phosphorylated p65 translocates from the cytoplasm to the nucleus in NaCl-treated astrocytes (Figure 3A–3C).

Matrix metalloprotease-9 (MMP-9) is involved in the homeostasis of the extracellular matrix, and participates in angiogenesis by releasing VEGF [23]. We found that the MMP-9 and VEGF expression was significantly increased under high-salt conditions (Figure 3A, 3B, 3D). By using the SN50 inhibitor of p65 nuclear translocation, we found that SN50 decreased the protein expression of VEGF and MMP9 (Figure 3E), indicating that HSD induces VEGF and MMP-9 through the p65 NF-κB-dependent pathway.

### Astrocyte-derived VEGF is the main factor causing tight junction destruction in endothelial cells under high salt condition

**Figure 4:**
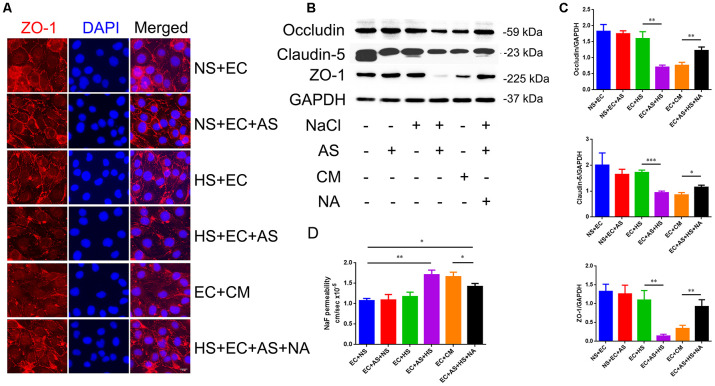
**Astrocyte-derived VEGF mediates HS-induced BBB breakdown.** (**A**) Representative double immunofluorescence staining of ZO-1^+^ bEnd.3 endothelium. Endothelial cells were cultured alone, or co-cultured with primary rats′ astrocytes, and treated with NS, HS, conditioned medium, or VEGF neutralizing antibody (NA). (**B**, **C**) Western blotting analysis of Occludin, Claudin-5, and ZO-1 in endothelial cells. (**D**) Permeability of tight junctions measured using NaF; *P<0.05, **P<0.01, compared with EC+NS. At least three separate experiments were conducted; means ± SD.

To determine whether the astrocyte-derived VEGF is involved in HS-induced tight junction destruction, mice brain microvascular endothelial cells, bEnd.3, were co-cultured with primary rat astrocytes in a transwell culture plate divided into 6 groups: 1. Endothelial cells cultured alone in normal medium (NS+EC group); 2. Endothelial cells co-cultured with astrocytes in normal medium (NS+EC+AS group); 3. Endothelial cells cultured in high-salt medium (40mM NaCl for 24h) alone (HS+EC group); 4. Endothelial cells co-cultured with astrocytes in high-salt medium (HS+EC+AS group); 5. Astrocytes pre-treated with 40mM NaCl(24h), and cultured with endothelial cells in conditioned medium (EC+CM group); 6. VEGF neutralizing antibodies (NA; 0.1μg/mL) added into the HS+EC+AS group, and cultured 24h (HS+EC+AS+NA group). Immunofluorescence staining was performed to analyze the morphology of ZO-1, one of the TJ proteins. As shown in Figure 4A, the structure of ZO-1 was intact and normal when endothelial cells were exposed to NaCl directly. However, the expression of ZO-1 was destroyed when endothelial cells were co-cultured with astrocytes in high salt conditions, or treated with conditioned medium from high salt-cultured astrocytes. Western blotting showed that the expression of occludin, claudin-5, and ZO-1 decreased in the HS+EC+AS and EC+CM groups (Figure 4B, 4C). Analysis of the permeability of tight junctions using NaF showed similar results to western blotting (Figure 4D).

### Effect of VEGF on tight junction is mediated via ERK and eNOS pathway

**Figure 5:**
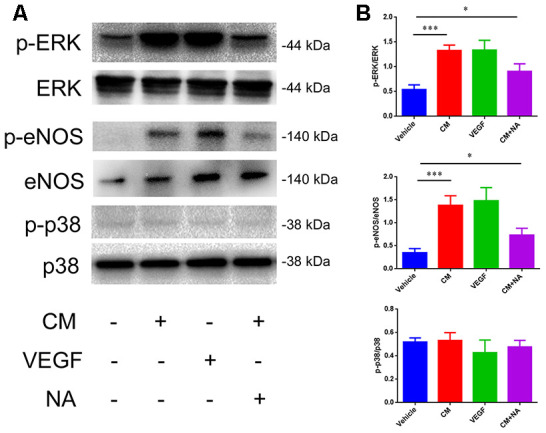
**Effect of VEGF on tight junction is mediated via ERK/eNOS.** (**A**, **B**) Protein levels of ERK/pERK, eNOS/pSer^1177^, eNOS, and p38/p-p38 in endothelial cells; *P<0.05, **P<0.01, compared with vehicle. At least three separate experiments were conducted; means ± SD.

Next, we analyzed the molecular mechanism involved in the NaCl-mediated down-regulation of TJ protein expression. It has been reported that high-salt conditions increase eNOS phosphorylation, and activate thep38/MAPK pathway [24, 25]. Moreover, VEGF activates PLCγ, which stimulates MEK-MAPK and p38in endothelium, and induces expression of eNOS via PLCγ and PI3K [26]. Thus, we examined the phosphorylation status of ERK, eNOS, and p38, which represents the degree of activation, by western blotting. As shown in Figure 5, a dramatic up-regulation of p-ERK/ERK and p-eNOS/eNOS levels was observed in endothelial cells treated with conditioned medium from high salt-cultured astrocytes, or with VEGF recombinant rat protein. Anti-VEGF neutralizing antibody (NA) partially reversed the up-regulation of p-ERK/ERK and p-eNOS/eNOS levels.

### VEGF^hi/+^ mice exhibit increased cerebral microvascular permeability

**Figure 6:**
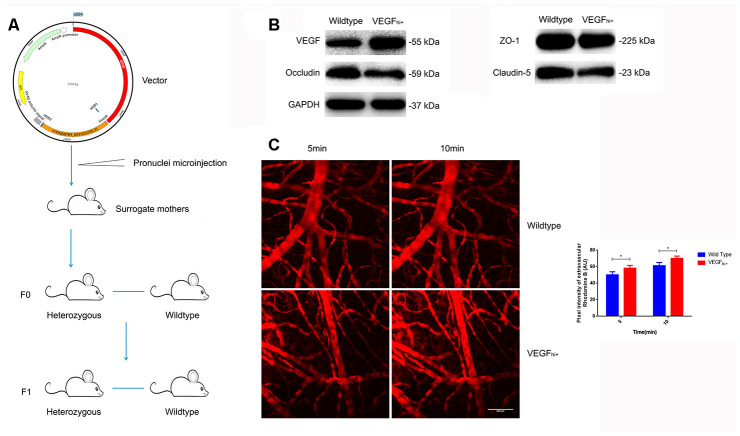
**Increased cerebral microvascular permeability in VEGF^hi/+^ mice.** (**A**) Schematic diagram of VEGF^hi/+^ mice generation. (**B**) Immunoblotting showing VEGF, Occludin, Claudin-5 and ZO-1expression in VEGF^hi/+^ and wildtype mice. (**C**) Cerebral cortical micro-vessel and dye leakage at 5 and 10 min after injection of Rhodamine B isothiocyanate-dextran; n=5, *P<0.05, **P<0.01, compared with NSD. At least three separate experiments were conducted; means ± SD.

To corroborate the above results, we constructed VEGF^hi/+^ mice with up-regulated astrocyte-derived VEGF by transferring a plasmid with GFAP promoter and mVegfa open reading frame into a fertilized egg (Figure 6A). The VEGF^hi/+^ mice showed no significant changes in the expression of ZO-1, but a down-regulation of occludin and claudin-5 (Figure 6B). In addition, in the VEGF^hi/+^ mice cortical, the fluorescent dye solution leaked from microvessels significantly more than in the wildtype mice (Figure 6C).

### Blocking VEGF attenuates HSD-induced disruption of tight junction

**Figure 7:**
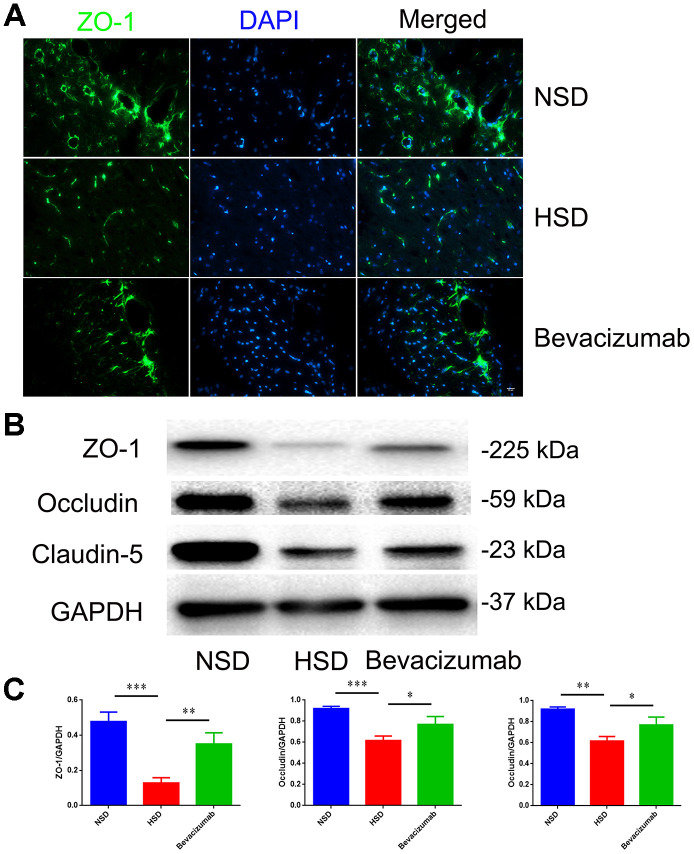
**Blocking VEGF attenuates disruption of tight junctions induced by HSD.** (**A**) Representative images showing double immunofluorescence staining of ZO-1 in green and DAPI in the brain specimens of rats. (**B**, **C**) Expression of Occludin, Claudin-5 and ZO-1 determined by western blotting; n=5, *P<0.05, **P<0.01, compared with NSD. At least three separate experiments were conducted; means ± SD.

Next, we tested if blocking VEGF using the bevacizumab antibody could reduce the destruction of tight junctions induced by HSD. Rats in the HSD group were intraperitoneally injected with10 mg/kg bevacizumab or an equal volume of normal saline (continuous administration for 30days). We found that the HSD-induced disruption of tight junction proteins in the rat brain could be attenuated by blocking VEGF with bevacizumab (Figure 7A–7C).

## DISCUSSION

Although salt is an essential nutrient for maintaining water-electrolyte balance, excessive salt intake causes a range of health problems [27]. It has long been known that HSD leads to various cerebral and systemic vascular diseases resulting from hypertension [28, 29]. However, a recent study showed that HSD promotes neurovascular and cognitive dysfunction and exacerbates BBB disruption in permanent cerebral ischemia independently of its effects on arterial pressure [30]. Our HSD rat model proves that there are no alterations in systolic blood pressure compared to normal diet controls. Since recent studies have shown that HSD promotes the onset of CNS autoimmune diseases, such as multiple sclerosis [31], we speculate that high salt increases cerebral vascular permeability, leading to infiltration of peripheral immune cells into CNS. This hypothesis is supported by recent studies showing that CNS-infiltrating immune cells participate in pathophysiological processes under high salt conditions [7].

Most previous studies have focused on the short term effects induced by salt. However, HSD can also induce various vascular pathological manifestations in a long-term. Since we found no significant changes in tight junction proteins in the HSD rats observed for 30 days, we adjusted the time of high-salt feeding to 180 days to better mimic sustained high salt intake in humans. Using this chronic model of HSD, we found a marked reduction in tight junction proteins and a significant functional impairment of the BBB. Two-photon microscopy observation of increased microvascular exudation in the cerebral cortex of HSD mice further confirmed microvascular injury. Previous studies have demonstrated that the excess salt collects in the interstitial area in the body [32]. Astrocytes are the most widely distributed mesenchymal cells in the brain, and also an important structure of BBB, which is surrounded by their end-feet [33]. We have found previously that astrocytes can respond quickly to changes in the microenvironment, secreting a range of inflammatory factors and mediators to participate in a variety of pathophysiological processes [34]. Our previous results have also demonstrated that high salt induces astrocyte activation and secretion of various inflammatory factors [16]. The neuroimmune function of astrocytes is often neglected because microglia are usually thought to be only antigen presenting cells in the brain. However, recent studies have demonstrated that astrocytes can not only act as a regulator of the microglial inflammatory response, but also play an important role in regulating leukocyte effector functions [35].

Recent studies have suggested that astrocyte-derived VEGF drives BBB disruption in CNS inflammatory disease [26]. Interestingly, our previous studies have shown that high salt up-regulates VEGF expression in astrocytes at both gene and protein levels, independently of osmotic pressure [16]. The release of VEGF is known to activate the classic NF-κB/MMP-9 axis, which plays a key role in regulating the cellular immune response to stimuli, such as stress and cytokines [36]. Our data demonstrate that the NF-κB/MMP-9/VEGF pathway is activated in astrocytes under high NaCl conditions, indicating that NaCl may stimulate the inflammatory response.

Increasing evidence suggests that up-regulation of VEGF is closely related to the blood-brain barrier injury [37]. Li et al. have shown that neurons disrupt the endothelial barrier by activating astrocytes to up-regulate VEGF when subjected to ischemic injury [38]. Argaw et al. have reported that astrocyte-derived VEGF is the initiating factor for the destruction of the blood-brain barrier in CNS inflammatory diseases [26]. However, astrocytes are not the only source of VEGF. Endothelial cells themselves can also secrete VEGF. Thus, we first analyzed the direct effect of high salt on the tight junction between endothelial cells; however, it had a little effect. Then we constructed an *in vitro* cell model by co-culturing primary rat astrocytes and bEnd.3 to simulate the blood-brain barrier. Interestingly, when endothelial cells were co-cultured with astrocytes under high salt conditions, the expression, function, and morphology of tight junction proteins in endothelial cells significantly altered. These findings indicate that high salt induces astrocytes to release substance(s) that lead to the destruction of the endothelial barrier. Moreover, since a similar effect was observed when a high-salt astrocyte-conditioned medium was added, and anti-VEGF neutralizing antibody attenuated the aforementioned effect, our data indicate that the substance is the astrocyte-derived VEGF. VEGF^hi/+^ mice further confirmed the effect of VEGF by exhibiting increased cerebral microvascular permeability and decreased expression of TJ proteins.

Mitogen-activated protein kinase (MAPK) signal transduction pathways are involved in a variety of biological processes, such as proliferation, differentiation, transformation, and apoptosis. There are three parallel MAPK signaling pathways in mammalian cells: the extracellular signal-regulated kinase (ERK) signaling pathway; c-Jun N-terminal kinase (JNK) pathway, and p38/MAPK pathway [39]. These MAPK signaling pathways have different biological effects in the human body. Activation of the p38/MAPK/SGK1 pathway is inextricably linked to the cellular effects of high salt [5]. It was reported that excess salt exacerbates the blood-brain barrier disruption via a p38/MAPK/SGK1-dependent pathway in permanent cerebral ischemia [24]. In addition, HSD increases inhibitory nitric oxide synthase (eNOS) phosphorylation to inhibit the production of nitric oxide (NO) resulting in a decrease in cerebral blood flow in mice [25]. Our results indicate that the effect of VEGF is mediated through the activation of the ERK/eNOS pathway, which is consistent with previous studies. However, since the anti-VEGF neutralizing antibody could not completely reverse the impairment *in vivo* and *in vitro*, our results indicate that other substances might be involved; this necessitates further studies.

In summary, our results show that the astrocyte-derived VEGF increases cerebral microvascular permeability under high salt conditions. Our data indicate that the HSD-induced VEGF is dependent on p65/NF-κB pathway, and activates the ERK/eNOS pathway, resulting in the disruption of tight junction proteins. These findings suggest a potential risk role of the HSD-induced, astrocyte-derived VEGF, which can compromise the brain microenvironment and alter the BBB function.

## MATERIALS AND METHODS

### Animal experiments

8-week-old specific pathogen-free male Sprague-Dawley rats (weighing 250-280g), 1-day-oldspecific pathogen-free Sprague-Dawley rats, and 8-week-old male C57BL/6 mice weighing 20–25 g) were obtained from the experimental animal center of Sun Yat-sen University, and kept in a pathogen-free room. Transgenic male mice overexpressing VEGF against a C57BL/6 background were obtained from Cyagen Biosciences, Inc (Suzhou, China). The animal use protocols were reviewed and approved by the Institutional Animal Care and Use Committee of Sun Yat-sen University (Guangzhou, China).

In the rat experiment, we randomly assigned 40 male Sprague-Dawley rats aged 8 weeks to 2 groups. Group 1 received normal chow and tap water (normal salt diet, NSD) for 30 and 60 days. Group 2 received a sodium-rich chow containing 8% NaCl and 1% saline (high salt diet, HSD) for 30and 180 days. Group 2 was divided into two sub-groups: the intervention group receiving 10 mg/kg bevacizumab (Avastin, 25mg/ml, Roche, USA) at day 150, or the control group receiving an equal volume of saline solution intraperitoneally injected for the remaining 30 days.

In the mice experiment, we randomly assigned 10 C57BL/6 aged 8 week-old mice to LSD (< 0.1% NaCl) and tap water, or HSD (4% NaCl) and 1% saline. After mice had been on their specified diets for 90 days, they were used in two-photon imaging experiment.

### Transgenic vector

We inserted the Glial fibrillary acidic protein (GFAP) promoter and mVegfa open reading frame in a regular plasmid to construct a gene expression vector. By pronuclear microinjection, we injected the constructed vector into the fertilized egg, which was then implanted into a surrogate mother to obtain the desired offspring through germline transmission.

### Cell culture

Primary astrocytes were isolated from 1-day-old Sprague-Dawley rats as described before [16, 40]. The immortalized murine brain microvascular endothelial cell line bEnd.3 (ATCC, Manassas, VA, USA) was used for the *in vitro* assay. Cells were cultured alone or co-cultured in transwell plates. All experiments were conducted using 80%–85% confluent cells. In the high salt group, the plated cells were incubated with serum-free DMEM medium for1 h, followed by a 24 h incubation in serum-free DMEM containing 40 mM NaCl (Sigma-Aldrich). Recombinant VEGF (CST, #5211), anti-VEGF neutralization antibody (R&D, #AF564) or SN50 (MCE, #213546-53-3) were added into the medium as indicated.

### In vivo two-photon imaging

Mice were anesthetized, operated on to construct a thin cranial window (3 mm in diameter), fixed on a custom-fabricated metal frame and placed under a two-photon laser scanning microscope (Leica, Germany)equipped with a water-immersion objective lens (25×). Data acquisition and laser scanning were performed using Leica Application Suite Advanced Fluorescence 2.5 software, at a wave length of 860 nm. To monitor the cerebral microvascular permeability using detection of leaked dyes, Rhodamine B isothiocyanate-dextran (1.4% in saline, 70 kDa molecular weight, Sigma-Aldrich) was injected intravenously to visualize the brain vasculature. We selected the red fluorescence channel for detection, and calculated the average fluorescence intensity in the extravascular compartment. Images of the XYZ stacks (512 × 512 pixels) were collected to a depth of 200 μm (2-μm step size) below the cortical surface, at 5and 10 min after the injection. We defined the vessels with a diameter of 20-40um as microvascular.

### Evans blue dye (EBD) extravasation

In brief, a 4% solution of EBD (4 ml/kg of body weight) was injected intraperitoneally and allowed to circulate for 2 hours at day 180 before execution. Under deep anesthesia, rats were perfused with saline until colorless fluid outflowed from the right atrium. Then, ischemic cerebral hemispheres were collected after decapitation. The brain specimens were weighed (wet weight of each sample was 50 mg), homogenized in 1 ml of 50% trichloroacetic acid, and centrifuged at 15,000× g for 20 minutes. Then, 0.5 ml of the resultant supernatant was added to 1.5 ml of anhydrous ethanol for a colorimetric assay using a fluorescence spectrophotometer (Ex620 nm, Em680 nm) to determine the EBD concentration. The EBD content (per mg of wet weight) within the brain tissue was used to determine the BBB permeability rate of EBD.

### Noninvasive blood pressure measurement

The computerized tail-cuff system (BP2010A, sofron, China) was used to perform the non-invasive systolic blood pressure measurement. Measurements were conducted at day 180before execution.

### Immunofluorescence staining

Frozen sections were incubated with control 1.5% goat serum for 30 min, and then stained using mouse monoclonal anti-CD31 (1:100, Thermo Fisher, #MA1-81051), rabbit polyclonal anti-ZO1 (1:100, Thermo Fisher, #40-2200), mouse monoclonal anti-GFAP (1:1000, CST, #3670), rabbit polyclonal anti-VEGF (1:100; Millipore, #07-1420), mouse monoclonal anti-p65 (1:1000, CST, #6956), or rabbit monoclonal anti-p-p65 (1:1000, CST, #3033) primary antibodies, followed by fluorescent conjugated secondary antibody Alexa Fluor 647goat anti-mouse IgG (1:400, Thermo Fisher, A32728) or Alexa Fluor 488-conjugated goat anti-rabbit IgG (1:400, Thermo Fisher, A32731).

Cells were plated on poly-L-lysine (PLL)-treated cover slips, fixed in 4% paraformaldehyde for 20 min, and blocked with 10% goat serum in PBS. Slides were incubated overnight in a humidified chamber at 4 °C with the following primary antibodies: rabbit polyclonal anti-AQP4 (1:200, CST, Thermo Fisher, #PA5-77716) or rabbit polyclonal anti-ZO1 (1:100, Thermo Fisher, #40-2200). After primary antibody incubation, samples were washed, and incubated with fluorescent secondary antibody Alexa Fluor 647-conjugated goat anti-rabbit IgG (1:400, Thermo Fisher, A32733)for 1 hour. The cells were counterstained by DAPI. Images were captured with a fluorescence microscope (IX71, Olympus).

### Western blot analysis

Cells and brain tissues were homogenized with RIPA buffer containing protease inhibitor cocktail (Sigma). Supernatants were collected by centrifugation at 12,000 g for 20 min at 4°C. The concentration of total protein in each sample was quantitated by BCA method (Thermo). Equal amounts of proteins were separated by SDS-PAGE, and then transferred to membranes (Immobilon-P; Millipore) for 1 hour. The membranes were blocked for 1 hour and incubated with the following primary antibodies: mouse monoclonal anti-GFAP (1:1000, CST, #3670), rabbit polyclonal anti-VEGF (1:1000; Millipore, #07-1420), rabbit polyclonal anti-ZO1 (1:1000, Thermo Fisher, #40-2200), mouse monoclonal anti-Occludin (1:1000, Thermo Fisher, #33-1520), rabbit polyclonal anti-Claudin5 (1:1000, Thermo Fisher, #PA5-37527), mouse monoclonal anti-MMP9 (1:500, Thermo Fisher, #MA5-14228), mouse monoclonal anti-p65 (1:1000, CST, #6956), rabbit monoclonal anti-p-p65 (1:1000, CST, #3033), rabbit monoclonal anti-p38 (1:1000, CST, #8690), rabbit monoclonal anti-p-p38 (1:1000, CST, #4511), rabbit polyclonal anti-ERK (1:1000, CST, #9102), rabbit polyclonal anti-p-ERK (1:1000, CST, #9101), rabbit polyclonal anti-eNOS (1:1000, CST, #9572), or rabbit polyclonal anti-p-eNOS^thr495^ (1:1000, CST, #9574) antibody in blocking solution at 4°C overnight. After triple washing, the membranes were incubated with the corresponding secondary antibody (1: 5000) at room temperature for 1 hour. In order to confirm equal protein loading, the blots were also incubated with antibodies against GAPDH (1:5000, CST, #8884). The target protein was detected using the ECL-Plus kit (Millipore), and ImageJ software was used to analyze the relative protein densities.

### Measuring the permeability of co-cultured cells by using NaF

NaF was used for permeability studies as described before [41]. Astrocyte/endothelial cells co-cultured in the transwell plate were used to measureapical to basolateral (A to B) transport of NaF. About 200mL of fresh pre-warmed transport buffer (141 mM NaCl, 4 mM KCl, 2.8 mM CaCl2, 1 mM MgSO4, 10 mM HEPES, and 10 mM D-glucose, pH 7.4) containing 250 mM NaF were added to the apical chamber and 800 mL of fresh transport buffer were added to the basolateral chamber. Cells were incubated in a humidified atmosphere (5%CO_2_/95% air) at 37°C for 1 hour. At the end of incubation period, the buffer from both chambers was separately collected. NaF concentrations in apical and basolateral compartments were determined by measuring their fluorescence intensities at excitation/emission wavelengths of 720 and 529 nm, respectively (multi-plate reader BIO-TEK, Winooski, VT, USA). Apparent permeation coefficient (*Pc*, cm/sec) was calculated from the following equation: *P_c_*(cm/sec)=*V_b_*×*C_b_*/ *C_a_*×*A*×*T*. *V_b_* is the volume of basolateral side (800 mL), *C_b_* is the concentration of NaF (mM) in the basolateral side, *C_a_* is the concentration of NaF (mM) in the apical side, A is the membrane area (0.33 cm^2^), and T is the time of transport (3,600 sec).

### Quantitative real-time PCR

Total RNA was extracted using TRIzol (Life Technologies), and reverse transcription was performed from 3 μg total RNA using oligo (dT) and RevertAid Reverse Transcriptase (Thermo Scientific) according to the supplier’s instructions. Quantitative PCR was performed with SuperReal PreMix SYBR Green (TIANGEN) using an Applied Biosystems 7500 Fast Real-Time PCR System (Life Technologies). Relative cDNA level was calculated by the comparative CT (cycle threshold) method. PCR primers were the following (5′ to 3′): VEGF Sense: GTGAGCCAGGCTGCAGGAAG, Antisense: GAATGCGTCTGCCGGAGTCT, GAPDH Sense: ATGATTCTACCCACGGCAAG, Antisense: CTGGAAGATGGTGATGGGTT.

### Statistical analysis

Data are shown as mean ± SD. One-way analysis of variance (ANOVA) was performed on datasets using SPSS 13.0. For each end point, the treatment means were compared using the Bonferroni least significant difference procedure. A *P* value <0.05 was considered significant.

### Ethics statement

This study was approved by the ethics committee of the Third Affiliated Hospital of Sun Yat-sen University, and all participants signed informed consent forms.

The animal study was carried out in accordance with the recommendations of the animal use protocol, which was approved by the Institutional Animal Care and Use Committee of Sun Yat-Sen University. No potential conflicts of interest were disclosed.
